# Diet and risk of Barrett’s oesophagus: Melbourne collaborative cohort study

**DOI:** 10.1017/S0007114522002112

**Published:** 2023-04-14

**Authors:** Sabrina E. Wang, Allison Hodge, S Ghazaleh Dashti, Suzanne C. Dixon-Suen, Natalia Castaño-Rodríguez, Robert Thomas, Graham Giles, Alex Boussioutas, Bradley Kendall, Dallas R. English

**Affiliations:** 1 Centre for Epidemiology and Biostatistics, Melbourne School of Population and Global Health, University of Melbourne, Melbourne, VIC, Australia; 2 Cancer Epidemiology Division, Cancer Council Victoria, Melbourne, VIC, Australia; 3 Clinical Epidemiology and Biostatistics Unit, Murdoch Children’s Research Institute, Melbourne, VIC, Australia; 4 Department of Paediatrics, University of Melbourne, Melbourne, VIC, Australia; 5 Institute for Physical Activity and Nutrition, Deakin University, Geelong, VIC, Australia; 6 School of Biotechnology and Biomolecular Sciences, University of New South Wales, Kensington, NSW, Australia; 7 Department of Medicine, Royal Melbourne Hospital, Melbourne, VIC, Australia; 8 Precision Medicine, School of Clinical Sciences at Monash Health, Monash University, Clayton, VIC, Australia; 9 Department of Gastroenterology, The Alfred, Melbourne, VIC, Australia; 10 Central Clinical School, Monash University, Melbourne, VIC, Australia; 11 Department of Medicine, The University of Queensland, Brisbane, QLD, Australia; 12 Department of Gastroenterology and Hepatology, Princess Alexandra Hospital, Brisbane, QLD, Australia

**Keywords:** Vegetable, Fruit, Dietary fibre, Carotenoids, Barrett’s oesophagus, Gastroesophageal reflux, Oesophageal adenocarcinoma

## Abstract

Barrett’s oesophagus (BE) is the precursor of oesophageal adenocarcinoma, which has become the most common type of oesophageal cancer in many Western populations. Existing evidence on diet and risk of BE predominantly comes from case–control studies, which are subject to recall bias in measurement of diet. We aimed to investigate the potential effect of diet, including macronutrients, carotenoids, food groups, specific food items, beverages and dietary scores, on risk of BE in over 20 000 participants of the Melbourne Collaborative Cohort Study. Diet at baseline (1990–1994) was measured using a food frequency questionnaire. The outcome was BE diagnosed between baseline and follow-up (2007–2010). Logistic regression models were used to estimate OR and 95 % CI for diet in relation to risk of BE. Intakes of leafy vegetables and fruit were inversely associated with risk of BE (highest *v*. lowest quartile: OR = 0·59; CI: 0·38, 0·94; *P*-trend = 0·02 and OR = 0·58; CI: 0·37, 0·93; *P*-trend = 0·02 respectively), as were dietary fibre and carotenoids. Stronger associations were observed for food than the nutrients found in them. Positive associations were observed for discretionary food (OR = 1·54; CI: 0·97, 2·44; *P*-trend = 0·04) and total fat intake (OR per 10 g/d = 1·11; CI: 1·00, 1·23), the association for fat was less robust in sensitivity analyses. No association was observed for meat, protein, dairy products or diet scores. Diet is a potential modifiable risk factor for BE. Public health and clinical guidelines that incorporate dietary recommendations could contribute to reduction in risk of BE and, thereby, oesophageal adenocarcinoma.

Barrett’s oesophagus (BE) is a premalignant metaplastic condition of the distal oesophagus and the precursor to oesophageal adenocarcinoma^(1)^. Incidence of both BE and oesophageal adenocarcinoma has been rising in Western populations^(2–4)^. Major risk factors for BE and oesophageal adenocarcinoma include gastroesophageal reflux disease (GERD) and adiposity^(1,5)^, and diet is a modifiable risk factor for both conditions. We previously identified that while some dietary compositions might affect risk of developing GERD, adherence to a diet that has been associated with lower cancer risk and mortality, as reflected by the Mediterranean Diet Score^(6,7)^ or the Alternative Healthy Eating Index-2010^(8)^, did not appear to reduce the risk of GERD^(9)^.

A systematic review and meta-analysis based on three cohort studies by the World Cancer Research Fund/American Institute for Cancer Research in 2017 reported that consumption of vegetables (risk ratio [RR] = 0·89; CI: 0·80, 0·99 per 100 g/d) and green leafy vegetables (RR = 0·85; CI: 0·74, 0·96 per 50 g/d) reduced risk of oesophageal adenocarcinoma^(10)^. No conclusive evidence was found for other dietary factors^(10)^. A recent meta-analysis based on three case–control studies reported that dietary fibre intake was associated with lower risk of BE (highest *v*. lowest category: OR = 0·42; CI: 0·29, 0·61)^(11)^.

Results from case–control studies might be affected by recall bias because cases and controls are likely to report their diet differently. A prospective study design where diet is measured before onset of BE could overcome this bias. Only one large cohort study has examined diet and risk of BE. The Netherlands Cohort Study reported that vegetable intake was associated with reduced BE risk for males but not for females^(12)^, and no association was observed for meat intakes^(13)^. There a paucity of evidence on diet and risk of BE in other populations, which might differ due to difference in dietary patterns and prevalence of GERD and BE. We thus conducted a comprehensive analysis to investigate the potential effect of diet, including macronutrients, carotenoids, food groups, specific food items, beverages and dietary scores, on risk of BE in a culturally diverse cohort.

## Subjects and methods

### Study participants

The Melbourne Collaborative Cohort Study (MCCS) is a cohort of 41 513 participants. In addition to participants born in Australian, the cohort intentionally targeted recruitment of people born in Italy and Greece to broaden the range of observations of measured lifestyle factors including diet^(14)^. Participants aged 40–69 years were recruited through the electoral roll between 1990 and 1994. The study protocol was approved by the Human Research Ethics Committee at Cancer Council Victoria (CCV IEC 9001). Written consent to participate was obtained on recruitment. For this study, participants older than 63 years at baseline were excluded as they were not followed-up for BE outcomes. We further excluded participants with history of cancer (except for keratinocyte skin cancers), diabetes mellitus or CVD at baseline as they likely had changed their diet following diagnosis; those whose total energy intake was deemed implausible (in the top or bottom 1 % of total energy intake); and those who had missing data for diet or any identified confounders (detailed below) at baseline. A total of 28 504 participants were eligible. A *post hoc* exclusion was applied to participants who were diagnosed with BE before baseline (*n* 3), based on diagnosis date collected at follow-up.

### Measurement of diet at baseline

Information on diet at baseline was collected using a self-administered 121-item FFQ developed specifically for the MCCS^(15)^. Food items were selected for inclusion in the questionnaire based on results of weighed food records from 810 volunteers of similar demographic background to the MCCS participants. Items were included if they contributed to the first 80 % of any nutrient for at least one of the sex-specific country of birth stratum (Australia, Italy or Greece). Intake for each food item was reported as one of nine frequencies, from never or less than once per month to six or more times per day. The estimated frequencies for food groups were calculated by converting each of the nine food frequencies to a daily value and summing across items^(15)^. The ‘discretionary food’ group included foods containing ‘high saturated fat’ or ‘added sugars’ as suggested by the Australian Dietary Guidelines^(16)^, which included the following items from the questionnaire: ice cream, sweet biscuits, cakes or sweet pastries, puddings and chocolate confectionary. To calculate nutrient intakes, sex-specific portion sizes were allocated to each item based on the weighed food record data. Evaluation studies within the MCCS suggest diet was reasonably measured with moderate correlation between several nutrient intakes and their plasma concentrations^(17,18)^.

We selected dietary factors that have been associated with risk of gastroesophageal reflux symptoms^(9,19)^, BE^(12)^ or oesophageal adenocarcinoma^(5,10)^. We also investigated two diet scores, the Mediterranean Diet Score and the Alternative Healthy Eating Index-2010. We used a modified version of the Mediterranean Diet Score from Trichopoulou *et al.* ^(7)^ Briefly, one point each was assigned for intake above the sex-specific medians for vegetables, fruit, cereal, legumes and fish; one point each was assigned for intake below the medians for dairy and red meat; one point was assigned for daily alcohol intake between 10–50 g/d for men and 5–25 g/d for women; and the ninth point was assigned based on olive oil intake^(6)^. A score of 9 indicates the highest degree of adherence. The Alternative Healthy Eating Index-2010 scores diet based on consumption frequency of foods and nutrients that are predictive of chronic diseases risk, including: vegetables, fruit, whole grains, sugar-sweetened beverages and fruit juice, nuts and legumes, red or processed meat, trans fat, long-chain fats, polyunsaturated to saturated fat ratio, sodium and alcohol^(8)^. A higher score predicts lower risk of chronic diseases, with 110 being the highest score.

Information on demographic and other lifestyle factors was also collected at baseline via structured interviews^(14)^, and anthropometric measures, including height and weight, were measured.

### Ascertainment of Barrett’s oesophagus

Information on gastroesophageal reflux and BE was collected via telephone interviews between 2007–2010. Participants were asked if they had ever been diagnosed with BE by a doctor. If so, information was collected on when the diagnosis was made, and details of the treating doctor. For all participants who said they had been diagnosed or did not know if they had, attempts were made to obtain copies of relevant endoscopy and pathology reports and correspondence from gastroenterologists and endoscopists to the participants’ usual medical practitioners.

BE cases were defined an endoscopic diagnosis of columnar-lined oesophagus. If an endoscopy report was not available, an oesophageal biopsy showing columnar epithelium or correspondence from a gastroenterologist stating endoscopically diagnosed BE was used. We used the definition endoscopically confirmed columnar epithelium for BE to prevent misclassifying intestinal metaplasia identified from biopsy taken from a regular or irregular Z line as BE. Diagnoses of BE were reviewed by a gastroenterologist (BJK). The primary BE definition is consistent with the British Society of Gastroenterology guidelines^(20)^. We additionally examined BE cases restricted to those with specialised intestinal metaplasia diagnosed from an oesophageal biopsy as a secondary definition, which is consistent with the American College of Gastroenterology^(21)^ and Australian guidelines^(22)^.

### Statistical analyses

Logistic regression was used to estimate OR and CI for dietary variables in relation to risk of BE. As the risk of BE was rare (0·9 % in our eligible cohort), the OR is a good approximation of the risk ratio^(23)^. Macronutrients and carotenoids were analysed as continuous variables. Macronutrients were energy-adjusted using the residual method^(24)^. For example, the energy-adjusted fat intake is the residuals from a regression of fat intake on total energy intake. Increments reported are based on approximately one sd of intake. Food groups, food items and beverages were analysed as approximate quartiles of frequency (times/d) using the lowest quartile as the reference group. The Mediterranean Diet Score was analysed using predefined categories (score 1–3, 4–6, 7–9) and the Alternative Healthy Eating Index-2010 was analysed as quartiles, both using the least adherent category as the reference. Tests for linear trend for food intake and diet score were performed using the median in each category. Tests for linearity assumption were performed using likelihood ratio tests comparing models with each dietary variable fitted as a categorical *v*. a pseudo-continuous variable.

All analyses included potential confounders identified from a causal diagram based on the literature (online Supplementary 1). The potential confounders included: age, sex, country of birth (Australia/New Zealand/Northern Europe, Italy or Greece), an area-based measure of socio-economic position (the Index of Relative Socioeconomic Disadvantage from the Socio-economic Indexes for Areas^(25)^), educational attainment (primary school or less, high/technical school or tertiary), smoking status (never, former or current), physical activity score (four categories from least to most active) and average lifetime alcohol intake (g/d).

Our primary analysis assumed that the association between diet and BE was the same for males and females. We performed a secondary analysis stratified by sex as existing literature suggests sex may be a potential effect modifier^(9,12)^. Test for interaction between diet and sex was performed by including an interaction term in analysis models and using likelihood ratio tests.

With a sample of 20 793 participants, an average BE risk of 0·9 % and a reference group made up with one-quarter of the sample, a minimum OR of 1·5 or 0·67 can be detected with 80 % power and two-sided significance level of 0·05.

### Sensitivity analyses

#### Further adjustment for dietary confounders

The observed effect of one dietary factor on risk of BE could be due to low intake of another inversely correlated dietary factor. For example, an apparent effect of low vegetable intake on BE risk could either truly be attributable to vegetable deficiency in diet, or it could be due to high fat intake, which is often inversely correlated with vegetable intake. We thus performed a sensitivity analysis further adjusting for dietary confounders that were inversely correlated with each dietary exposure. To avoid collinearity in the regression model, we examined the Pearson correlation for dietary factors (online Supplementary 2). The strongest inverse correlation included in analysis models was between total carbohydrate and total meat intake (*r* = −0·57). For the analysis of fat and protein, the models additionally included total vegetable and total fruit; for the analysis of carbohydrate and fibre, the models additionally included total meat intake; for the analysis of meat intake, the model additionally included total carbohydrate and total fibre; for the analysis of carotenoids, vegetable and fruit, the model additionally included total fat intake as a dietary confounder. The analysis of dairy, discretionary food, chocolate, carbonated beverages, tea and coffee were not further adjusted for dietary confounders, as their intake was not strongly correlated with other dietary factors (all correlation coefficients < 0·26).

### Further adjustment for adiposity

The primary analysis assumed adiposity to be a mediator (i.e., on the causal pathway from diet to risk of BE; online Supplementary 1). In general, it is more likely that diet influences adiposity risk. However, it is also plausible given the age of our cohort participants that adiposity affected diet at baseline. In this case, adiposity would be a confounder (i.e., a common cause of exposure and outcome). We thus performed a sensitivity analysis further adjusting for adiposity, measured as BMI.

### Further adjustment for *H. pylori* infection

*H. pylori* infection could affect appetite-regulating hormones^(26)^, which, in turn, could have reduced dietary intake at baseline. *H. pylori* infection has also been associated with lower risk of BE^(27)^. We were unable to include *H. pylori* infection status as a confounder in our primary analysis as it was not measured for all participants. To assess the potential confounding impact of *H. pylori* infection on our findings, we repeated the primary analyses and further adjusted for *H. pylori* infection in a random subset of participants with *H. pylori* data from a previous case–control study nested in the MCCS (*n* 1311). *H. pylori* antibodies were measured in baseline plasma using an immunoblotting kit (Helicoblot 2·1; Genelabs Diagnostics, Singapore).

### Assessing potential bias from gastroesophageal reflux symptoms at baseline

The analysis included some participants who reported gastroesophageal reflux symptoms before baseline. This was to ensure that the distribution of reflux symptoms among BE cases was representative of the distribution in the target population (around two-thirds of BE patients reported reflux symptoms before their diagnosis^(28)^). However, participants with symptoms before baseline might have changed their diet as a mean to alleviate symptoms, which means diet measured at baseline might not accurately reflect their average diet. To investigate how this measurement error in diet might have affected our results, we performed a sensitivity analysis restricted to participants without reflux symptoms at baseline. As information on reflux symptoms was collected at follow-up, there were missing data on symptom status (*n* 218) and time of symptom onset (*n* 732) for some eligible participants. Missing data on reflux symptoms were multiply imputed using chained equations, methods are described in Supplementary 3^(29)^. We excluded participants born in Italy or Greece from this sensitivity analysis because few BE cases from this group (*n* 2) reported symptoms before baseline.

In addition, for those who reported ever having reflux symptoms ≥ 1 d/week, we compared dietary intakes at baseline for those who had symptom onset before *v*. after baseline as a proxy to examine how participants with symptoms might have changed their diet.

### Assessing potential selection bias from loss to follow-up

There is risk for selection bias when characteristics of participants lost to follow-up are different from those who completed follow-up (online Supplementary 4). One way to minimise this selection bias is by including participant characteristics related to lost to follow-up in the analysis models (online Supplementary 4A). We included most of the demographic and lifestyle factors related to lost to follow-up in the main analysis models, as they were also identified as potential confounders for the association between diet and BE. We did not however include adiposity in the analysis models, as it was identified as a potential mediator (i.e., on the causal path) between diet and risk of BE. As BMI was slightly higher in those who did not complete follow-up in our study, there was risk for selection bias. To examine the potential impact of this selection bias on our findings, we compared the predicted probability of completing follow-up for BMI 20 kg/m^2^, 25 kg/m^2^ and 30 kg/m^2^ at baseline using logistic regression models that did and did not include other demographic and lifestyle factors as covariates. Similar predicted probability of completing follow-up across BMI values would suggest the impact of selection bias on our study results is small.

All analyses were performed using Stata version 16.

## Results

**Fig. 1.**
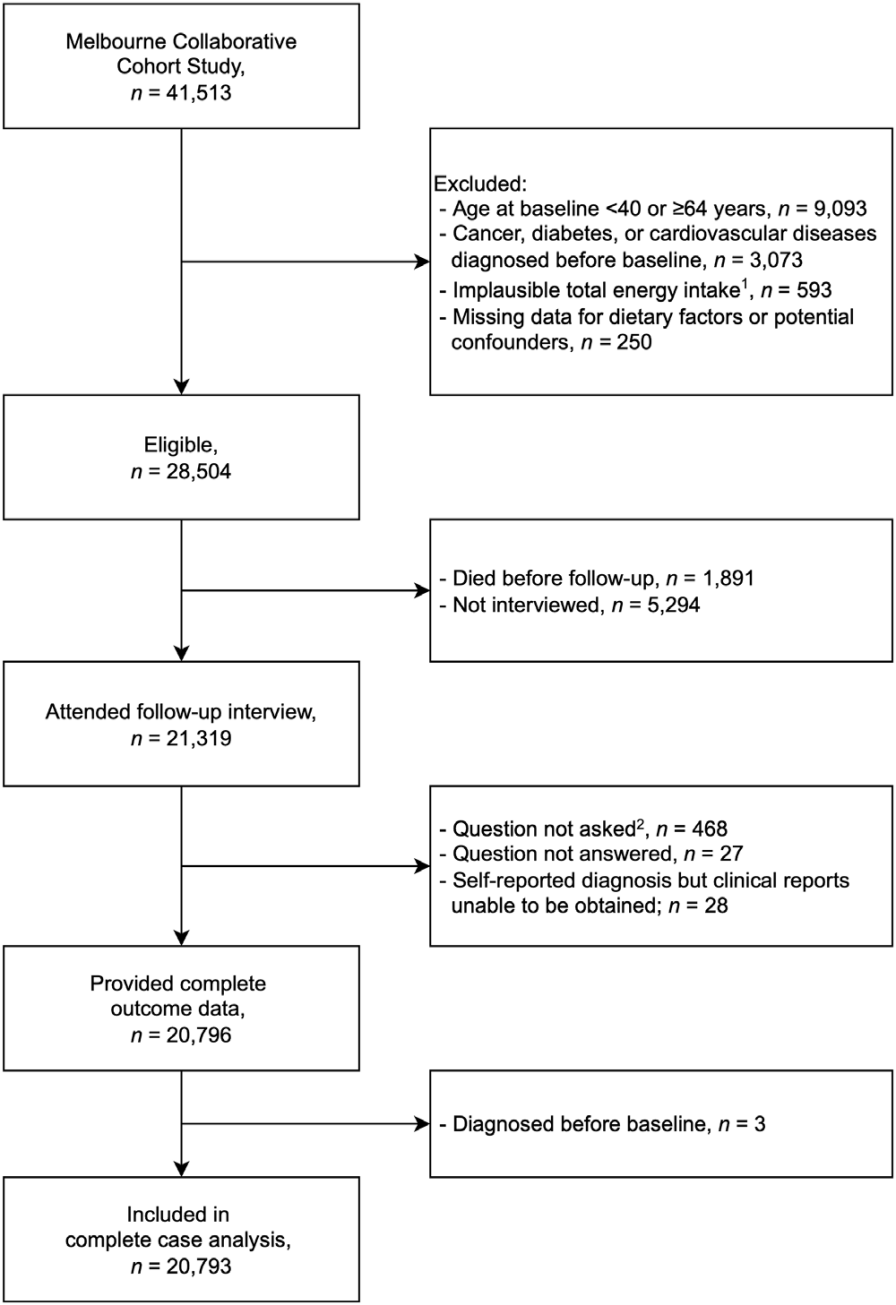
Participants flow diagram. Footnotes: ^1^Total energy intake in the 1st or 99th percentile. ^2^Version of the questionnaire did not contain questions on Barrett’s oesophagus.

Of the 28 504 eligible participants, 20 796 (73 %) attended follow-up and provided complete data (Fig. 1). Participants who did not complete follow-up were more likely to be older at baseline, born in Italy or Greece, more socioeconomically disadvantaged, with lower educational attainment, current smokers at baseline, less physically active, have had lower alcohol intake or higher BMI at baseline (online Supplementary 4). Distributions of dietary intake at baseline for those who did and did not complete follow-up were similar (online Supplementary 4). Three BE cases were excluded *post hoc* due to diagnosis before baseline, leaving 20 793 for analysis.

During a median follow-up of 16 years (range: 13–20 years), 193 participants (0·9 %) were diagnosed with BE, of whom 131 had confirmed specialised intestinal metaplasia. BE cases were more likely to be men, older, born in Australia/New Zealand/Northern Europe, less socioeconomically disadvantaged, former smokers or had higher BMI at baseline compared with the eligible cohort (Table 1). Those diagnosed with BE consumed less fruit at baseline compared with the eligible cohort (Table 2).

**Table 1.** Baseline characteristics of Barrett’s oesophagus cases and total eligible participants in the Melbourne Collaborative Cohort Study (Numbers and percentages)

	BE cases (*n* 193)		Total eligible (*n* 20793)	
	*n*	%	*n*	%
Sex				
Male	103	53·4	7853	37·8
Female	90	46·6	12940	62·2
Age				
44–44	39	20·2	4755	22·9
45–49	41	21·2	4649	22·4
50–54	43	22·3	4416	21·2
55–59	37	19·2	4075	19·6
60–63	33	17·1	2898	13·9
Median, years	51·9		51·1	
IQR	46·6, 57·9		45·4, 57·0	
Country of birth				
AU/NZ/Northern Europe	180	93·3	16963	81·6
Italy	8	4·1	2197	10·6
Greece	5	2·6	1633	7·9
Socio-economic position*				
Q1 (most disadvantaged)	23	11·9	3110	15·0
Q2	26	13·5	3821	18·4
Q3	29	15·0	3372	16·2
Q4	46	23·8	4073	19·6
Q5	69	35·8	6417	30·9
Education				
Some high/technical school	80	41·5	10327	49·7
High/technical school	60	31·1	4444	21·4
Tertiary	53	27·5	6022	29·0
Cigarette smoking				
Never	100	51·8	12640	60·8
Former	77	39·9	6067	29·2
Current	16	8·3	2086	10·0
Physical activity level†				
Q1 (least active)	27	14·0	4468	21·5
Q2	47	24·4	4231	20·3
Q3	66	34·2	6748	32·5
Q4	53	27·5	5346	25·7
Alcohol intake‡				
Never	40	20·7	5134	24·7
Former	18	9·3	2318	11·1
Low	113	58·5	10892	52·4
High	22	11·4	2449	11·8
BMI				
<25 kg/m^2^	64	33·2	8552	41·1
25–< 30 kg/m^2^	88	45·6	8626	41·5
≥ 30 kg/m^2^	41	21·2	3615	17·4
Median, kg/m^2^	27·0		25·9	
IQR	24·4, 29·2		23·4, 28·7	

AU, Australia; BE, Barrett’s oesophagus; IQR, interquartile range; NZ, New Zealand.

* In quintiles of socio-economic position.

† In quartiles of physical activity level.

‡ Low intake for male < 40 g/d and female < 20 g/d; high intake for male ≥ 40 g/d and female ≥ 20 g/d.

**Table 2.** Baseline diet for Barrett’s oesophagus cases and total eligible participants in the Melbourne collaborative cohort study (Mean values and standard deviations)

	BE cases		Total eligible	
	*n* 193		*n* 20 793	
	Mean	sd	Mean	sd
Mean nutrient intake (sd), g/d				
Total fat	84	13	82	14
Saturated fat	34	7	33	7
Monounsaturated fat	29	5	29	6
Polyunsaturated fat	14	4	13	4
Total protein	98	15	98	15
Total carbohydrate	244	32	248	37
Starch	123	27	121	27
Sugar	120	36	125	40
Total fibre	30	8	31	8
Vegetable/fruit fibre	13	7	15	8
Cereal fibre	12	5	12	5
Alpha-carotene	2·1	1·2	1·9	1·3
Beta-carotene	5·8	2·8	5·7	3·1
Beta-cryptoxanthin	0·33	0·29	0·38	0·32
Lutein and zeaxanthin	3·3	1·7	3·7	2·2
Lycopene	7	4·8	7·9	5·0
Mean food group intake (sd), times/d				
Total meat	1·7	0·8	1·7	0·8
Red meat	1·1	0·6	1·1	0·7
Fish	0·3	0·2	0·3	0·2
Chicken	0·3	0·2	0·3	0·3
Processed meat	0·4	0·3	0·4	0·4
Total vegetable	5	2·5	5·5	3·1
Leafy vegetable	0·9	0·6	0·8	0·7
Cruciferous vegetable	0·5	0·4	0·7	0·6
Total fruit	3·5	2·7	4·2	3·1
Total dairy products	5·4	3·0	5	2·8
Discretionary food	1·5	1·5	1·3	1·3
Mean food item intake (sd), times/d				
Citrus	0·8	0·8	1	0·9
Tomato	0·3	0·3	0·4	0·5
Chocolate	0·2	0·4	0·2	0·4
Carbonated beverage	0·3	0·6	0·4	0·8
Tea	2·2	1·9	1·8	1·8
Coffee	1·8	1·7	2	1·7
Mean dietary score (sd)				
Mediterranean Diet Score	4	2	4	2
Alternate Healthy Eating Index-2010	64	11	65	11

BE, Barrett’s oesophagus; sd, standard deviation.

**Fig. 2.**
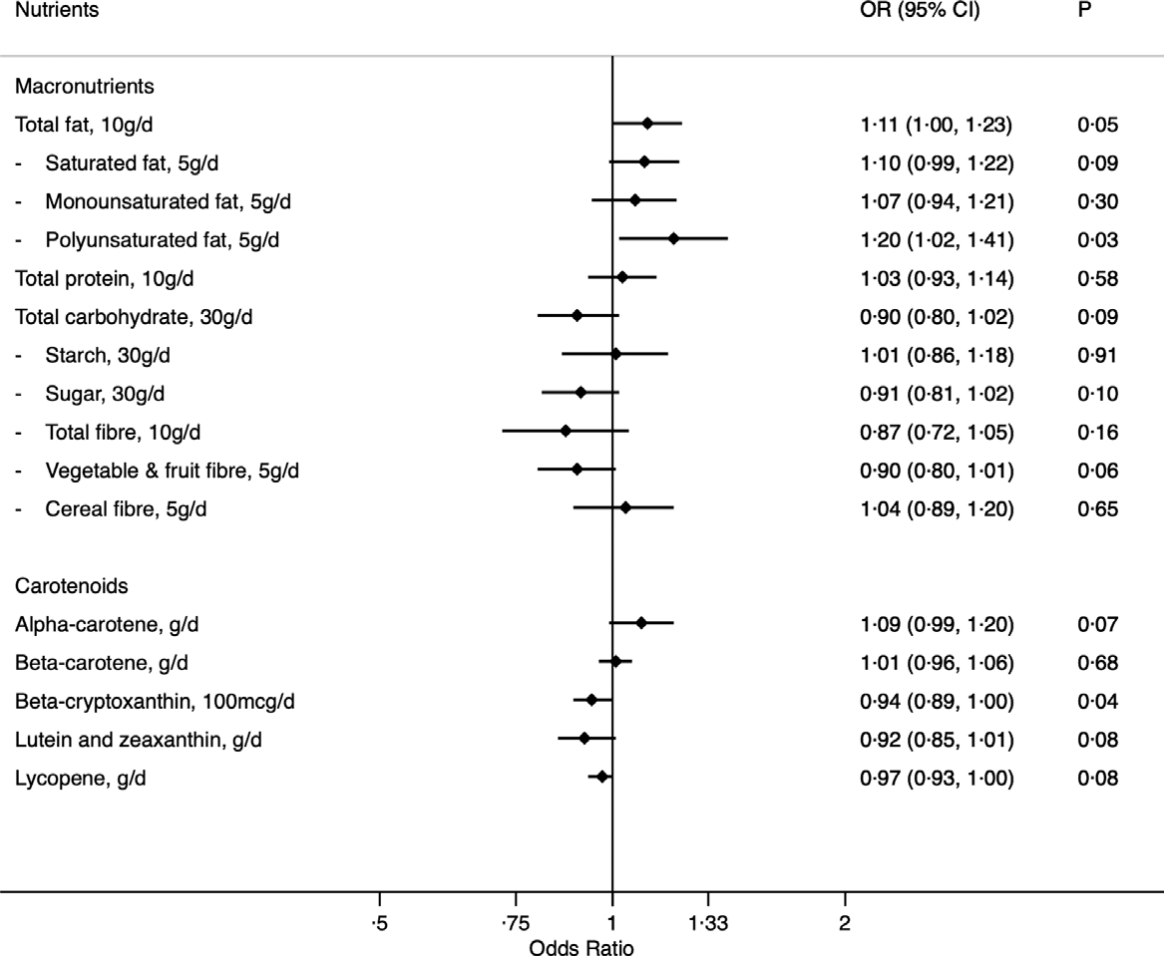
OR for nutrient intakes in relation to risk of Barrett’s oesophagus. Footnotes: OR estimated from analysis models including age, sex, country of birth, socio-economic position, educational attainment, smoking status, physical activity score and average lifetime alcohol intake as covariates.

OR for nutrient intakes in relation to risk of BE are presented in Fig. 2. Fat intake (OR = 1·11 per 10 g/d; CI: 1·00, 1·23; *P*-trend = 0·05) was positively associated with BE risk with the strongest association observed for polyunsaturated fat (OR = 1·20 per 5 g/d; CI: 1·02, 1·41; *P*-trend = 0·03) compared with other types of fat. Vegetable and fruit fibre (OR = 0·90 per 5 g/d; CI: 0·80–1·01; *P*-trend = 0·06), beta-cryptoxanthin (OR = 0·94 per 100 mcg/d; CI: 0·89, 1·00; *P*-trend = 0·04), lutein and zeaxanthin (OR = 0·92 per g/d; CI: 0·85, 1·01; *P*-trend = 0·08) and lycopene (OR = 0·97 per g/d; CI: 0·93, 1·00; *P*-trend = 0·08) were weakly associated with lower risk of BE. There was also a weak positive association for alpha-carotene. No association was observed for protein or beta-carotene.

OR for food intake and adherence to diet scores in relation to risk of BE are presented in Table 3. For those in the highest quartile of intake for leafy vegetables (OR = 0·59; CI: 0·38–0·94; *P*-trend = 0·02), total fruit (OR = 0·58; CI: 0·37, 0·93; *P*-trend = 0·02), citrus (OR = 0·56; CI: 0·36, 0·87; *P*-trend = 0·01) and tomato (OR = 0·57; CI: 0·37, 0·87; *P*-trend = 0·02), the risk of BE was almost halved compared with those in the lowest quartile. Discretionary food (Q4 *v*. Q1 OR = 1·54; CI: 0·97, 2·44; *P*-trend = 0·04) and tea (Q4 *v*. Q1 OR = 1·51; CI: 1·00, 2·29; *P*-trend = 0·04) were positively associated with BE risk. No association was observed for meat, dairy, chocolate, carbonated beverage, coffee intake or the dietary scores.

**Table 3.** OR for food intakes and adherence to diet scores in relation to Barrett’s oesophagus (OR and 95 % CI)

	Quartiles of intake									
	Quartile 2		Quartile 3		Quartile 4		Increase of one time/d			
	OR	95 % CI*	OR	95 % CI*	OR	95 % CI*	OR	95 % CI*	Test for trend, *P*-value	Test for linearity, *P*-value†
Food group, Quartile 1 (ref)										
Total meat	0·90	0·60, 1·35	1·01	0·69, 1·48	0·89	0·56, 1·41	0·95	0·73, 1·25	0·723	0·799
Red meat	1·30	0·87, 1·93	1·10	0·73, 1·67	1·11	0·71, 1·74	1·03	0·75, 1·41	0·859	0·428
Fish	0·98	0·66, 1·44	1·13	0·78, 1·64	0·91	0·59, 1·41	0·85	0·31, 2·28	0·741	0·560
Chicken	1·11	0·77, 1·59	1·03	0·62, 1·70	1·13	0·75, 1·69	1·15	0·55, 2·40	0·706	0·848
Processed meat	0·96	0·63, 1·48	0·88	0·60, 1·28	0·92	0·61, 1·38	0·88	0·48, 1·64	0·694	0·581
Total vegetable	0·84	0·58, 1·20	0·75	0·49, 1·14	0·83	0·52, 1·32	0·97	0·90, 1·05	0·437	0·511
Leafy vegetable	0·67	0·48, 0·95	0·71	0·46, 1·09	0·59	0·38, 0·94	0·62	0·41, 0·93	0·021	0·367
Cruciferous vegetable	1·03	0·66, 1·59	1·11	0·73, 1·69	1·32	0·86, 2·03	1·25	0·92, 1·69	0·156	0·980
Total fruit	0·94	0·64, 1·38	0·75	0·51, 1·10	0·58	0·37, 0·93	0·89	0·81, 0·98	0·018	0·873
Total dairy products	1·16	0·76, 1·76	0·77	0·49, 1·22	1·29	0·84, 1·98	1·02	0·96, 1·09	0·505	0·045
Discretionary food	1·12	0·71, 1·74	1·15	0·73, 1·81	1·54	0·97, 2·44	1·18	1·00, 1·39	0·044	0·942
Food item, Quartile 1 (ref)										
Citrus	0·90	0·61, 1·32	0·93	0·63, 1·37	0·56	0·36, 0·87	0·75	0·60, 0·94	0·012	0·468
Tomato	0·76	0·50, 1·14	0·64	0·44, 0·92	0·57	0·37, 0·87	0·57	0·36, 0·90	0·017	0·290
Chocolate	1·24	0·85, 1·81	0·86	0·56, 1·32	0·90	0·59, 1·37	0·63	0·25, 1·62	0·342	0·195
Carbonated beverage	1·12	0·75, 1·67	1·21	0·83, 1·76	1·09	0·72, 1·64	1·04	0·70, 1·53	0·854	0·358
Tea	0·96	0·63, 1·46	0·87	0·57, 1·34	1·51	1·00, 2·29	1·10	1·01, 1·20	0·036	0·115
Coffee	0·99	0·67, 1·47	0·79	0·55, 1·13	0·78	0·51, 1·21	0·93	0·85, 1·03	0·152	0·764
Mediterranean Diet Score	Score 4–6		Score 7–9				Increase of one unit			
Score 0–3 (ref)	0·82	0·59, 1·15	1·03	0·61, 1·72			0·83	0·43, 1·62	0·590	0·194
Alternative Health Eating Index-2010	Quartile 2		Quartile 3		Quartile 4		Increase of one unit			
Quartile 1 (ref)	1·04	0·70, 1·56	0·97	0·64, 1·46	1·01	0·66, 1·55	1·00	0·89, 1·12	0·964	0·939

* OR estimated from logistic regression models including age, sex, country of birth, socio-economic position, educational attainment, smoking status, physical activity score and average lifetime alcohol intake.

† *P*-value from likelihood ratio test for departure from linearity.

When the outcome was BE defined as specialised intestinal metaplasia (*n* 131) (online Supplementary 5), the inverse associations for lutein and zeaxanthin, lycopene and leafy vegetables, and the positive association for discretionary food were stronger. The positive association for tea was no longer observed. A positive association was observed for cruciferous vegetables.

When the analysis was stratified by sex, there was no evidence for effect modification by sex (results not shown). The only exception was Mediterranean Diet Score (*P*–value for interaction = 0·03) but the CI for OR were wide for both men (OR = 0·44; CI: 0·18, 1·10) and women (OR = 2·28; CI: 0·62, 8·39).

### Sensitivity analyses

To examine the potential impact of confounding, we performed sensitivity analyses further adjusted for dietary confounders, BMI and *H. pylori* infection (in a subset of participants with *H. pylori* data) respectively, in addition to confounders already included. When further adjusted for dietary confounders (online Supplementary 6), the positive association for fat intake was attenuated; an inverse association was observed for total carbohydrate; the results for food groups, food items and diet scores were minimally changed. Further adjustment for BMI did not change the results markedly (online Supplementary 7). In a subset of participants with *H. pylori* data (*n* 1311), further adjusting for *H. pylori* infection did not change the results markedly (results not shown).

For sensitivity analysis that examined potential differential measurement error in diet due to reflux symptoms before baseline, there was no evidence for interaction between dietary factors and symptoms at baseline (results not shown). Results from analysis restricted to participants without reflux symptoms before baseline were similar to the primary analysis (Supplementary 8). For those who reported ever having symptoms ≥ 1 d/week, those who had onset before baseline had slightly higher intake of lycopene and lower intake of citrus at baseline compared with those who had onset after baseline (online Supplementary 9).

For sensitivity analysis examining the impact of selection bias from loss to follow-up, the predicted probability of providing complete BE data were more similar across BMI values after accounting for demographic and lifestyle factors (BMI 20 kg/m^2^ = 75 %; BMI 25 kg/m^2^ = 74 %; BMI 30 kg/m^2^ = 72 %) compared with without accounting for them (BMI 20 kg/m^2^ = 80 %; BMI 25 kg/m^2^ = 75 %; BMI 30 kg/m^2^ = 69 %).

## Discussion

Overall, vegetable and fruit fibre, beta-cryptoxanthin, lutein and zeaxanthin, lycopene, leafy vegetable, fruit, citrus and tomato intake reduced risk of BE, the inverse associations remained robust in most sensitivity analyses. Stronger associations were observed for fruits and leafy vegetables than for the nutrients found in them. The positive association for discretionary food remained robust in most sensitivity analyses, whereas the positive associations for fat, alpha-carotene and tea intake were less robust against sensitivity analyses.

Existing literature on diet and BE is mostly based on case–control studies, where diet is measured after diagnosis. It is possible that BE cases reported their diet differently from non-cases, and thus results may be affected by recall bias. A prospective study design ensures diet is measured in a disease-free cohort, thereby minimising the risk differential measurement error in diet. We were also able to account for key confounding factors, as well as investigate the robustness of our results under different assumptions on the underlying causal structure between diet and risk of BE. BE diagnosis was confirmed by a gastroenterologist (BJK) reviewing endoscopy and pathology reports, minimizing misclassification.

There are potential measurement errors, both random and systematic, in intake measured by the FFQ. Random errors may arise from inaccuracy in participant’s recall of their diet or difference in interpretation of the questionnaire items. Systematic errors may arise from the design of the FFQ. For example, the number of items included under each food group was different, with more items included for vegetables and fruits resulting in apparent higher intake. This limitation of FFQ has been pointed out in the nutritional epidemiology literature – the absolute intake measured is directly related to the number of questions^(30,31)^. However, the primary aim for the use of FFQ in our study was to rank people into quantiles of intake of foods and nutrients, rather than to accurately measure absolute intakes. Listing more items thus allowed more detailed analysis of nutrient composition than if items were further combined^(31)^. The systematic measurement error in absolute intake is unlikely to affect OR estimated based on categorised dietary variables (e.g., quartiles of intake), as the ranking is preserved^(24)^. However, OR based on increments of nutrient intake (e.g., g/d) may be underestimated due to systematic overestimation of intake caused by having more items under certain food groups (e.g., vegetables)^(24)^. This systematic error would be unequally distributed among participants^(24)^. Both systematic and random errors in measurement of diet would be non-differential as it is not affected by participant’s outcome for BE.

Some participants included in the analysis had reflux symptoms before baseline. This was to ensure that the study sample was representative of the target population in relation to distribution of reflux symptoms in BE cases^(28)^. However, this may have introduced differential error in measurement of diet, as participants with symptoms might have changed their diet for symptom alleviation. Our sensitivity analysis suggests the impact of this bias may be minimal, as there was no evidence for effect modification by symptom at baseline for the estimated OR for any dietary factor in relation to risk of BE.

Selection bias is possible as 27 % of the eligible participants were lost to follow-up and the distributions of demographic and lifestyle factors for those who did and did not provide complete BE data were different. However, given we have already accounted for most of the factors associated with completeness of follow-up in the analysis models, the impact of selection bias is minimised. From our sensitivity analysis, the impact from BMI, which is associated with completeness of follow-up but not included in the analysis models, is likely to be small after accounting for other pre-exposure demographic and lifestyle factors.

We performed a comprehensive analysis of the potential effect of diet on risk of BE, including dietary factors that have not been investigated in previous cohort studies on BE^(12,13)^. Consistent with Keszei *et al.*’s Netherlands Cohort Study^(12,13)^, we observed no association between meat intake and risk of BE. We also observed an inverse association for leafy vegetable intake but unlike Keszei *et al.* we did not observe an association for total, raw or Brassica vegetable intakes. Of all forms of vegetables studied, Keszei *et al.* observed the strongest association for raw leafy vegetable intake in males (HR = 0·55; CI: 0·36, 0·86)^(12)^. We did not observe effect modification by sex for the potential effect of vegetable intakes on risk of BE. In addition to leafy vegetable and total fruit, we observed inverse associations for vegetable and fruit fibre, beta-cryptoxanthin, lutein and zeaxanthin, lycopene, citrus and tomato intake. Cohort studies on oesophageal adenocarcinoma have reported inverse associations for green leafy vegetables^(32)^, raw vegetables^(33)^ and citrus fruits^(33)^, but not for total vegetable or fruit intake^(32–34)^. A recent meta-analysis of the three aforementioned studies^(32–34)^ reported a weak inverse association between vegetable intake and risk of oesophageal adenocarcinoma (RR = 0·89 per 100 g/d; CI: 0·80, 0·99)^(10)^. Another meta-analysis of four case–control studies reported beta-carotene was associated with reduced oesophageal adenocarcinoma risk^(35)^, but we did not observe an association for beta-carotene. We observed a positive association for ‘discretionary food’ as defined by the Australian Dietary Guidelines^(16)^. Dietary added sugar has been associated with increased risk of oesophageal adenocarcinoma in a cohort study (highest *v*. lowest quintiles: HR = 1·62; CI: 1·07, 2·45)^(36)^.

With results from our previous study on diet and risk of GERD in the MCCS^(9)^, we showed that GERD and BE may share some, but not all, dietary risk factors. For nutrients in relation to GERD, we observed sex-specific positive associations for fat and an inverse association for carbohydrate in men, but no associations with nutrients was observed for women. We also observed inverse associations for fruit, citrus and tomato and risk of GERD. We did not observe an inverse association between leafy vegetable and GERD. In contrast, we observed a positive association between cruciferous vegetables and GERD, which might be due to overlapping symptoms between irritable bowel syndrome and GERD^(9)^. In addition, carbonated beverages were associated with increased risk of GERD but not with BE. Diet scores were not associated with GERD or BE.

It is possible that any effect of diet on GERD is predominantly mechanistic. Both fat and carbonated beverage intake have been associated with increased transient relaxation of the lower oesophageal sphincter that leads to gastroesophageal reflux^(37,38)^. Conversely, the potential effect of diet on BE might be predominantly systemic. Dietary fibre has been associated with lower concentrations of inflammation biomarkers that promote carcinogenesis, such as interleukin-6 and tumour necrosis factor-*α* receptor-2^(39)^. An endoscopic study found that dietary fibre, but not fat intake, was associated with increased abundance of Firmicutes, the gram-positive bacteria that predominate in normal oesophagus, and with decreased abundance of gram-negative bacteria that predominate reflux oesophagitis and BE^(40)^. These gram-negative bacteria could trigger innate immune responses and subsequently induce chronic inflammation of the oesophageal lining^(41)^. In addition, *in vitro* and *in vivo* studies have suggested that carotenoids have antioxidant, antiapoptotic and anti-inflammatory properties that reduce risk of developing cancer^(42)^. It has been demonstrated *ex vivo* on forty-five BE tissues that oxidative stress and DNA damage can be induced by short exposure to low pH and bile acids^(43)^.

High dietary fibre intake could also reduce BE risk by reducing risk of gastroesophageal reflux and adiposity; both could mediate the effect of diet on risk of BE. Diets high in fibre may promote satiation, decrease macronutrients absorption and delay gastric emptying^(44)^. In our previous study on diet and GERD, there was a weak inverse association between fibre and risk of GERD in men^(9)^. In contrast, further adjusting for BMI did not remove the association between vegetable and fruit fibre and risk of BE in this present study.

We observed stronger associations for vegetables and fruits than for the nutrients found in them. This might be due to approximation in the calculation of nutrient intakes from the FFQ. It might also suggest fibre and carotenoids have synergistic effect on reducing risk of BE. The stronger association observed for food than nutrients could also be attributed to other phytonutrients found in vegetables and fruits. For instance, phytic acid found in high fibre food has been demonstrated to reduce cellular proliferation of BE cell lines *in vitro* ^(45)^.

Compared with case–control studies, our cohort study provided less biased estimates for the potential effect of diet on risk of BE. Dietary recommendations, particularly on increasing leafy vegetable and fruit intake, could be considered as a point of intervention in public health and clinical practice. Guidelines that incorporate dietary modifications could contribute to reduction in risk of BE and, thereby, oesophageal adenocarcinoma.
